# 吉西他滨联合铂类两药方案与单药方案对非小细胞肺癌治疗比较的*meta*分析

**DOI:** 10.3779/j.issn.1009-3419.2010.03.06

**Published:** 2010-03-20

**Authors:** 云久 苟, 玲娟 张, 启梅 杨, 蓉芳 张, 慧玲 郭, 雷 姜, 克虎 杨, 金徽 田

**Affiliations:** 1 730000 兰州，兰州大学循证医学中心 Evidence Based Medicine Center of Lanzhou University, Lanzhou 730000, China; 2 730000 兰州，兰州大学第二医院心胸外科， 730000 兰州，兰州大学第二临床医学院 The Second Clinical Medical College of Lanzhou University, Lanzhou 730000, China

**Keywords:** 肺肿瘤, 吉西他滨, *meta*分析, Lung neoplasma, Gemcitabine, *Meta* analysis

## Abstract

**背景与目的:**

吉西他滨联合铂类治疗晚期非小细胞肺癌的疗效是否优于单用吉西他滨或铂类尚存在争议，本研究对吉西他滨联合铂类治疗晚期非小细胞肺癌的有效性和安全性进行*meta*分析。

**方法:**

计算机检索VIP、CBM、CNKI、The Cochrane Library、PUBMED和EMBASE等数据库，同时追查纳入文献的参考文献和与本领域专家、通讯作者等联系以获取以上检索尚未发现的相关信息。收集吉西他滨联合铂类治疗晚期非小细胞肺癌的随机对照试验（RCT），根据Cochrane Handbook 5.0质量评价标准评价，用RevMan 5.0软件进行统计学分析。

**结果:**

共纳入4个随机对照试验（984名研究对象）的*meta*分析结果显示，吉西他滨联合铂类在有效率（OR=3.29, 95%CI: 1.79-6.05, *P*=0.000 1）、2年生存率（OR=3.22, 95%CI: 1.45-7.12, *P*=0.004）等方面与单用吉西他滨的差异有统计学意义，但联合用药组的不良反应发生率增加明显，以3/4级血小板减少发生率增加最为明显（RR=8.16, 95%CI: 1.71-39.07, *P*=0.009）；吉西他滨联合铂类在有效率（OR=3.51, 95%CI: 2.20-5.60, *P* < 0.01）、1年生存率（OR=1.67, 95%CI: 1.16-2.41, *P*=0.006）等方面与单用顺铂的差异有统计学意义，联合用药组的不良反应发生率增加明显，以3/4级血小板减少发生率增加最为明显（OR=28.55, 95%CI: 14.06-57.04, *P* < 0.01）。

**结论:**

与单药组相比，联合组可明显提高非小细胞肺癌患者的有效率和生存率，同时增加了毒副反应发生率。上述结果尚需高质量随机对照试验进一步证实。

## Abstract (en)

**Background and objective:**

Whether gemcitabine plus platinum chemotherapy is superior to gemcitabine or platinum single-agent chemotherapy for patients with non-small cell lung cancer (NSCLC) is still in dispute, and the aim of this study is to evaluate the efficacy and safety of gemcitabine combining platinum chemotherapy for patients with NSCLC.

**Methods:**

We searched relevant randomized controlled trials (RCTs) from VIP, CBM, CNKI, the Cochrane library, PUBMED and EMBASE. We traced the related references and experts in this field and communicated with other authors to obtain the information that has not been found. We made quality assessment of qualified RCTs assessed by the exclusion and inclusion criteria and used RevMan 5.0 provided by the Cochrane Collaboration to perform *meta*-analysis.

**Results:**

Four RCTs were eligible and included 984 patients. *Meta* analysis results suggested that: compared with gecitabine single-agent chemotherapy, the combination had a statistically significant benefit in increasing the response rate (OR=3.29, 95%CI: 1.79-6.05, *P*=0.000 1) and 2-year survival rate (OR=3.22, 95%CI: 1.45-7.12, *P*=0.004) while increased the risk of the incidence of adverse reactions, especially the grade 3-4 thrombocytopenia (RR=8.16, 95%CI: 1.71-39.07, *P*=0.009); compared with cisplatin single-agent chemotherapy, the combination had a statistically significant benefit in increasing the response rate (OR=3.51, 95%CI: 2.20-5.60, *P* < 0.01) and 1-year survival rate (OR=1.67, 95%CI: 1.16-2.41, *P*=0.006) while increased the risk of the incidence of adverse reactions, especially the grade 3-4 thrombocytopenia (OR=28.55, 95%CI: 14.06-57.04, *P* < 0.01).

**Conclusion:**

Compared with single-agent chemotherapy, the combining can significantly improve the efficiency and survival rate while increase the toxicity rare. The results still need to be proved by high quality RCTs.

据WHO统计，肺癌在全球癌症发病率和病死率中居第一位。2002年全球有118万人死于肺癌，占所有癌症患者死亡人数的17.6%^[1]^。2005年中国肺癌发病率比90年代初增加了1倍以上^[2]^。肺癌从治疗的角度可分为小细胞肺癌和非小细胞肺癌（non-small cell lung cancer, NSCLC），其中非小细胞肺癌约占肺癌的80%-85%，1/3患者初次确诊即为晚期^[3]^。以铂类为基础的化学治疗是目前晚期NSCLC的标准治疗方案。

吉西他滨（gemcitabine）是1996年美国上市的一种新型人工合成的嘧啶核苷类似物，进入人体后双氟脱氧嘧啶是其主要活性产物，主要作用于DNA合成后期和晚G_1_期，可以阻止细胞由G_1_期进入S期，抑制DNA链继续延长，并通过独特的掩蔽链干扰DNA链的自我修复机制，并可阻止RNA合成，导致细胞凋亡，从而引起细胞毒作用。同时，因吉西他滨固有的抑制DNA复制和修复的特征，适于与破坏DNA的药物联合^[4]^，而铂类药物的主要靶点正是增殖细胞的DNA。因此，吉西他滨联合铂类可增加铂类-DNA复合物的形成，并抑制DNA的修复，从而增加两药的协同作用^[5]^。目前，吉西他滨和铂类联合方案成为治疗晚期NSCLC的一线方案之一。然而有研究表明^[6, 7]^，吉西他滨联合顺铂对NSCLC的有效率为32%-54%，中位生存时间为8.4个月-15.1个月，血液学毒性和非血液学不良反应的发生率各为50%。而单用吉西他滨的有效率为31.5%，中位生存时间为7个月-9个月，毒副作用较温和，仅有10%-20%的患者发生血液学不良反应^[8-12]^。与单药方案相比，吉西他滨联合铂类方案在有效率、生存率上并无明显提高，不良反应发生率却大幅度增加，使NSCLC患者的生活质量因化疗药物毒性而受到影响。为了进一步证实吉西他滨联合铂类与吉西他滨或铂类单用治疗NSCLC的疗效和安全性，本研究采用Cochrane系统评价的方法对全世界相关随机对照试验进行评价，以期为临床决策提供参考依据。

## 1 资料与方法

### 1.1 纳入与排除标准

① 研究设计：随机对照研究；②研究对象：年龄≥18岁，经病理/细胞学检查证实的晚期NSCLC患者，治疗前4周内未接受过其他抗肿瘤治疗和无化疗禁忌证。排除伴有严重内科疾患及感染，同时伴随其他恶性肿瘤和肺癌为其他肿瘤转移病灶等；③干预措施：吉西他滨联合铂类*vs*铂类，吉西他滨联合铂类*vs*吉西他滨；④结局测量指标：有效率（部分应答率+全部应答率）、1年生存率、生活质量和不良反应等。

### 1.2 检索策略

计算机检索PubMed（1966-2009.10）、EMBASE（1974-2009.1）、Cochrane图书馆（2009年第1期）、维普数据库（1989-2009.1）、中国期刊全文数据库（1994-2008.10）、中国生物医学文献数据库（1978-2009.1）、万方数据库等中外文数据库。检索词包括：Non-small cell lung cancer、Non small cell lung carci- noma、Non small cell lung carcinomas、Non small cell lung、NSCLC、Gemcitabine、Carcinomas、非小细胞肺癌、吉西他滨、盐酸吉西他滨、泽菲、健择等。RCT检索策略遵循Cochrane系统评价手册5.0，其它检索采用主题词与自由词相结合的方式，并根据具体数据库调整，所有检索策略通过多次预检索后确定。另外，用Google Scholar、Medical martix等搜索引擎在互联网上查找相关的文献，追查已纳入文献的参考文献，与本领域的专家、通讯作者等联系，以获取以上检索未发现的相关信息。

### 1.3 文献筛选和资料提取

两位研究者交叉核对纳入研究的结果，对有分歧的意见通过讨论或由第3位研究者决定是否纳入。缺乏的资料通过电话或信件与作者联系予以补充。提取的信息资料主要包括：①一般资料：题目、作者姓名、发表日期、文献来源和参加中心数；②研究特征：研究对象的一般情况、各组病人的基线可比性及干预措施；③结局指标。

### 1.4 质量评价

按照Cochrane评价手册5.0评价RCT质量的评价标准，2位评价员独立对纳入研究的随机分配方法（分配方案隐藏、对研究对象、治疗方案实施者、研究结果测量者采用盲法）、结果数据的完整性（失访和退出）等进行质量评价。

### 1.5 统计学处理

采用Cochrane协作网提供的RevMan 5.0统计软件进行*meta*分析。计数资料采用相对危险度（risk rate, RR）或比值比（OR）为疗效分析统计量，计量资料采用加权均数差（W MD），各效应量均以95%CI表示。各纳入研究结果间的异质性采用*χ*^2^检验，若*P* > 0.1和*I*^2^ < 50%，采用固定效应模型进行分析，若异质性检验结果为*P* < 0.1和*I*^2^ > 50%时，分析异质性来源，确定是否能采用随机效应模型。如果研究间存在明显的临床异质性，只对其进行描述性分析，必要时采用敏感性分析检验结果的稳定性。

## 2 结果

### 2.1 文献检索结果

按照检索策略和资料收集方法（图 1），共查到相关文献326篇，通过排除重复或不符合纳入标准的文献，可能符合标准的文献有146篇，再经过阅读全文筛选出4项合格的随机对照试验。

**1:**
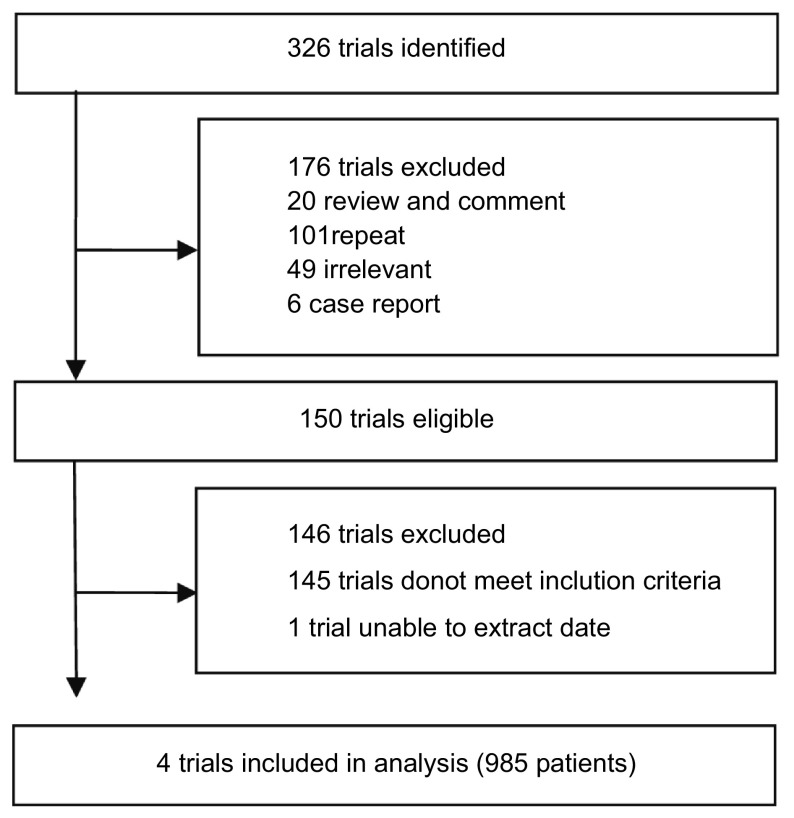
纳入研究流程图 Selection of trials

### 2.2 纳入研究基本特征

纳入的4项研究中，2项研究^[12, 13]^为来自17个不同国家和地区的多中心随机对照研究，3项研究^[13-15]^为吉西他滨联合铂类与吉西他滨治疗NSCLC的比较，1项研究^[12]^为吉西他滨联合铂类与顺铂治疗NSCLC的比较。4项研究^[12-15]^均报道了3/4级血液学及非血液学不良反应发生率，2项研究^[12, 13]^报道了有效率，2项研究^[14, 15]^仅报道了部分缓解率，3项研究^[12-14]^提到了生活质量评估，但无法得到具体数据（表 1）。3项研究^[12-14]^详细说明了随机方法，1项研究^[15]^未提及随机方法。2项研究^[12, 13]^正确实施分配隐藏，其余均未提及。2项研究^[12, 13]^采用双盲，其余均未提及。2项研究^[12, 13]^详细说明失访情况并进行了ITT分析（表 2）。

**1:** 纳入研究的一般特征 The characteristics of included studies

Included studies	Country	Intervations			Cases		Outcomes	Centers
		Experimental	Control		Experimental	Control		
Sandler 2000^[12]^	USA, Canada, England	G+Cisplatin	Cisplatin		260	262	a, b, c	17
Sederholm 2005^[13]^	Sweden	G+Carboplatin	G		164	170	a, b, c, d	17
Qing SUN 2006^[14]^	China	G+Cisplatin	G		22	23	b, c, e	1
ShujunLIU 2003^[15]^	China	G+Cisplatin	G		40	43	b, e	1
G：吉西他滨；a：有效率；b：毒副反应发生率；c：生活质量；d：疾病进展期；e：部分应答率。 G: gecitabine; a: efficacy rate; b: toxicity; c: the quality of life; d: time progression; e: partial response rate.								

**2:** 纳入研究的方法学质量 Quality assessment of methodology of included studies

Included studies	Randomization	Allocation concealment	Blinding	Withdral	ITT analysis
Sandler 2000^[12]^	Adequate	Adequate	Double-blind	Yes	Yes
Sederholm 2005^[13]^	Adquate	Adequate	Double-blind	Yes	Yes
Qing SUN 2006^[14]^	Adequate	Unclear	Unclear	Yes	Unclear
ShujunLIU 2003^[15]^	Unclear	Unclear	Unclear	Yes	Unclear

### 2.3 *meta*分析

#### 2.3.1 吉西他滨联合铂类*vs*吉西他滨

##### 2.3.1.1 有效率（图 2）

**2:**
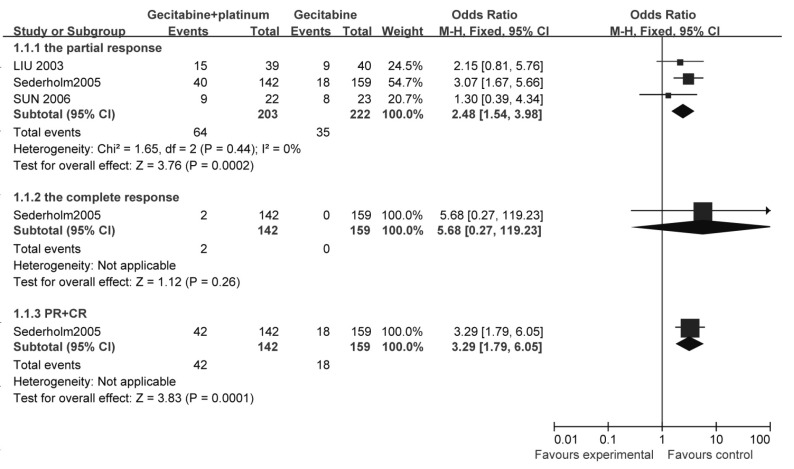
吉西他滨联合铂类与吉西他滨单药治疗NSCLC的有效率比较 The response rate of gecitabine+platinum versus gecitabine

1个研究^[13]^报告了有效率，结果显示：与单用吉西他滨相比，吉西他滨联合铂类可提高NSCLC患者有效率（OR=3.29, 95%CI: 1.79-6.05, *P*=0.000 1）。

3个研究^[13-15]^比较了部分应答率，各研究之间统计学异质性较小（*χ*^2^=1.65, *P*=0.44, *I*^2^=0%），采用固定效应模型计算合并后的综合效应。结果显示：与吉西他滨相比，吉西他滨联合铂类可提高部分应答率（OR=2.48, 95%CI: 1.54-3.98, *P*=0.000 2）。

##### 2.3.1.2 生存率（图 3）

**3:**
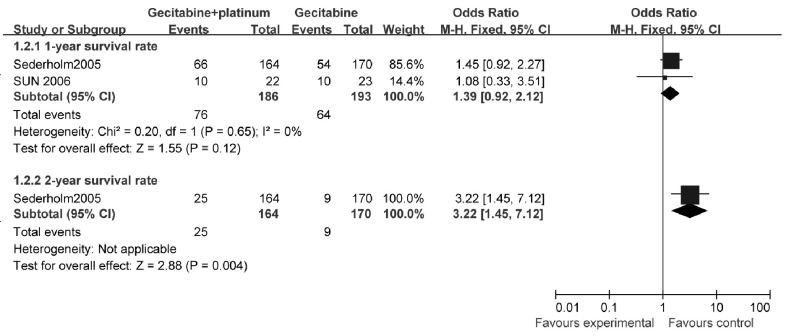
吉西他滨联合铂类与吉西他滨单药治疗NSCLC的生存率比较 The survival rate of gecitabine+platinum versus gecitabine

2个研究^[13, 14]^比较了1年生存率，研究之间统计学异质性较小（*χ*^2^=0.20, *P*=0.65, *I*^2^=0%），采用固定效应模型计算合并后的综合效应。结果显示：与单用吉西他滨相比，吉西他滨联合铂类治疗不能提高1年生存率（OR=1.39, 95%CI: 0.92-2.12, *P*=0.12）。

1个研究^[13]^报告了2年生存率，结果显示：与单用吉西他滨相比，吉西他滨联合铂类可提高2年生存率（OR=3.22, 95%CI: 1.45-7.12, *P*=0.004）。

##### 2.3.1.3 不良反应

###### 2.3.1.3.1 血液系统不良反应

3个研究^[13-15]^比较了3/4级血小板减少发生率，由于各研究之间存在异质性（*χ*^2^=3.61, *P*=0.16, *I*^2^=45%），采用随机效应模型计算合并后的综合效应。结果显示：与单用吉西他滨相比，吉西他滨联合铂类的血小板减少症发生率明显增加（RR=8.16, 95%CI: 1.71-39.07, *P*=0.009）（图 4）。

**4:**
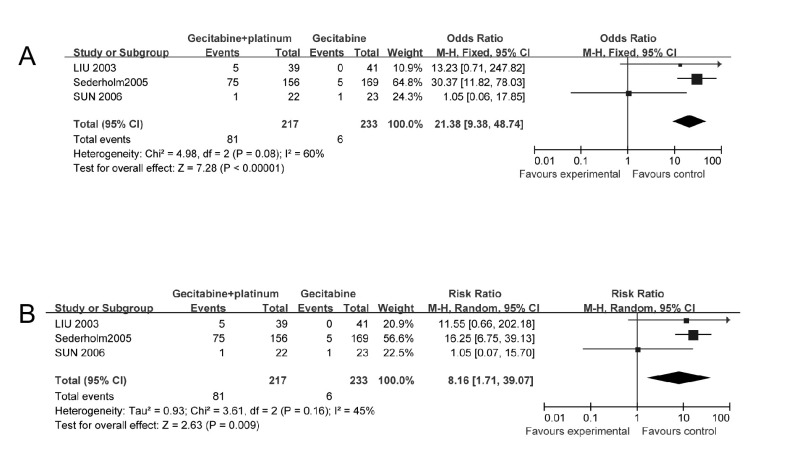
吉西他滨联合铂类与吉西他滨单药治疗NSCLC的生存率比较 The survival rate of Gecitabine+platinum versus Gecitabine

3个研究^[13-15]^报告了3/4级中性粒细胞减少发生率和3/4级贫血发生率，各研究之间异质性较小（*χ*^2^=0.58, *P*=0.75, *χ*^2^=0.24, *P*=0.89, *I*^2^均为0%），采用固定效应模型计算合并后的综合效应。结果显示：与单用吉西他滨相比，吉西他滨联合铂类的中性粒细胞减少发生率明显增加（OR=6.17, 95%CI: 3.35-11.33, *P* < 0.01），但不增加贫血的发生率（OR=2.51, 95%CI: 0.97-6.51, *P*=0.06）（图 5）。

**5:**
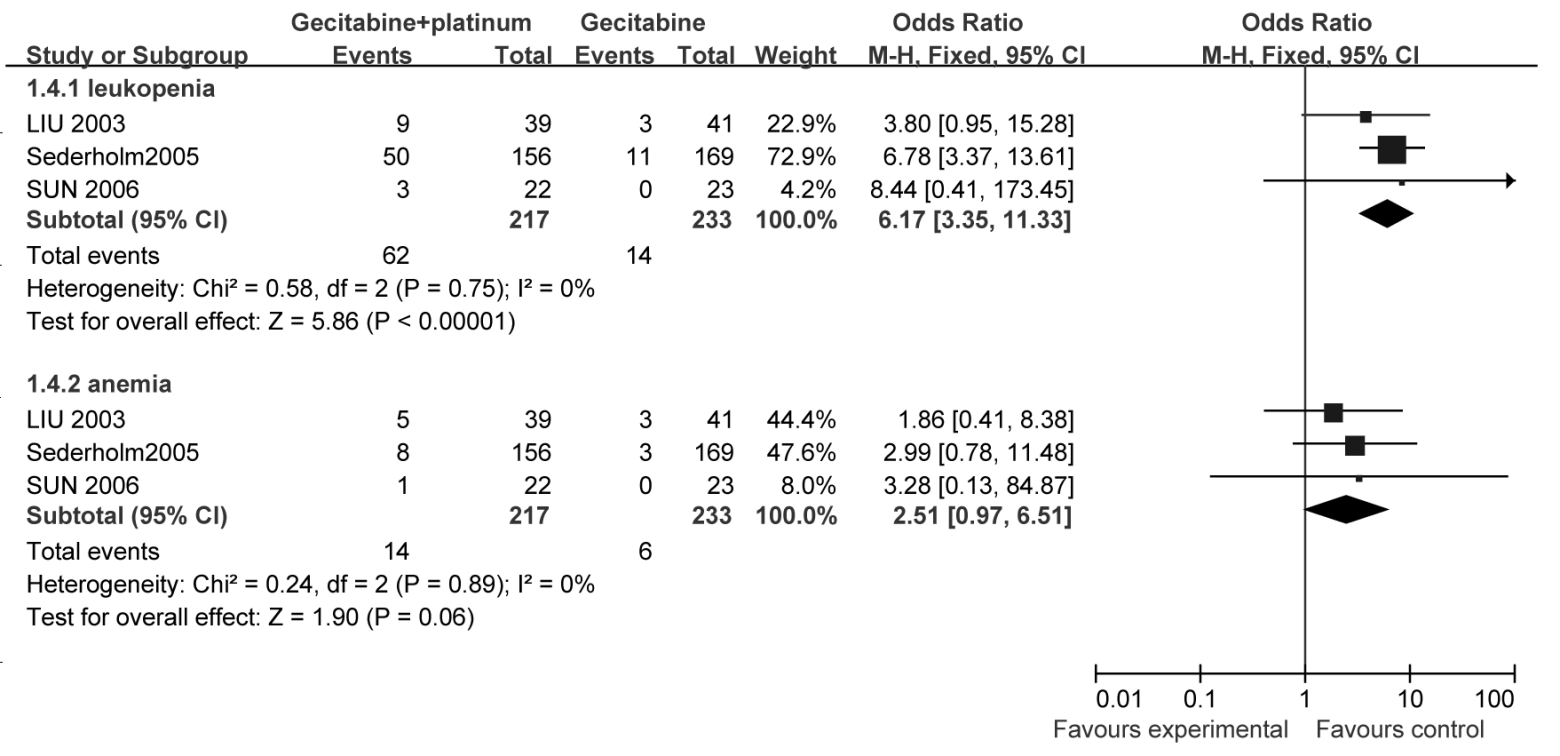
吉西他滨联合铂类与吉西他滨单药治疗NSCLC的中性粒细胞减少及贫血发生率 The grade 3-4 leukopenia and anemia of gecitabine+platinum versus gecitabine

###### 2.3.1.3.2 非血液系统不良反应（图 6）

**6:**
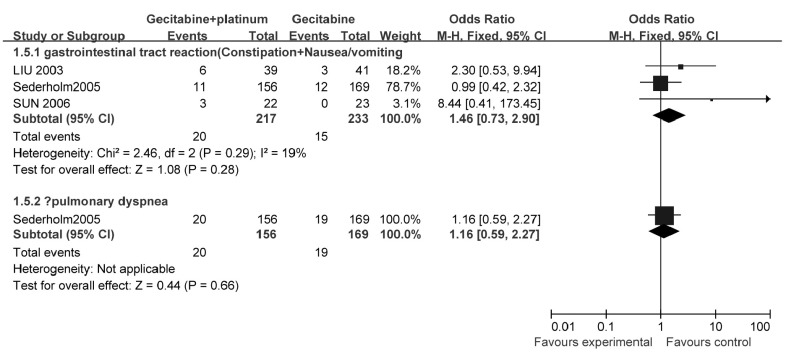
吉西他滨联合铂类与吉西他滨单药治疗NSCLC的非血液系统毒性发生率 The grade 3-4 unhemotolgical tocxity rate of gecitabine+platinum versus gecitabine

3个研究^[13-15]^报告了3/4级消化道不良反应（恶心/呕吐、便秘/腹泻）的发生率，各研究之间异质性较小（*χ*^2^=2.46, *P*=0.29, *I*^2^=19%），采用固定效应模型计算合并后的综合效应。结果显示：与单用吉西他滨相比，吉西他滨联合铂类不增加消化道不良反应发生率（OR=1.46, 95%CI: 0.73-2.90, *P*=0.28）。

1个研究^[13]^报告了肺相关不良反应/呼吸困难的发生率，结果显示：与单用吉西他滨相比，吉西他滨联合铂类不增加肺相关不良反应/呼吸困难的发生率（OR=1.16, 95%CI: 0.59-2.27, *P*=0.66）。

#### 2.3.2 吉西他滨联合铂类*vs*顺铂

##### 2.3.2.1 有效率

1个研究^[12]^报告了有效率，结果表明：与单用顺铂相比，吉西他滨联合铂类可明显提高NSCLC患者的有效率（OR=3.51, 95%CI: 2.20-5.60, *P* < 0.01）（图 7）。

**7:**
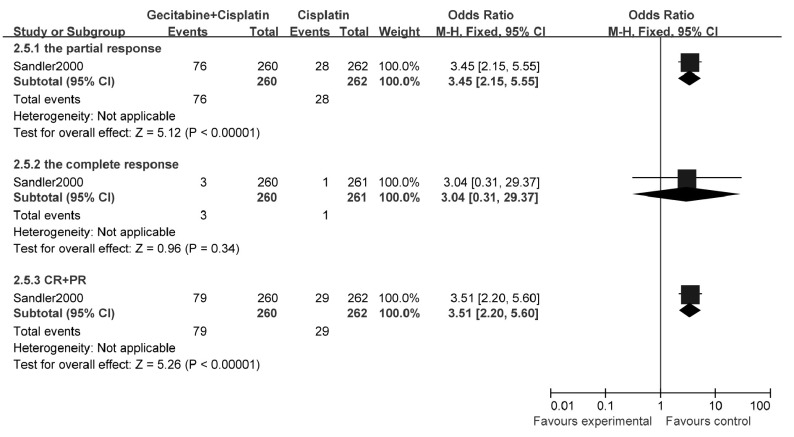
吉西他滨联合顺铂与顺铂治疗NSCLC的有效率 The response rate of gecitabine+cisplatin versus cisplatin

##### 2.3.2.2 生存率

1个研究^[12]^报告了1年生存率，结果表明：与单用顺铂相比，吉西他滨联合铂类可明显提高NSCLC患者的1年生存率（OR=1.67, 95%CI: 1.16-2.41, *P*=0.006）（图 8）。

**8:**
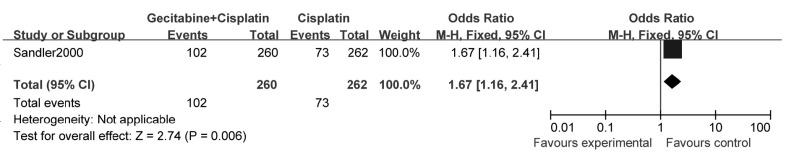
吉西他滨联合顺铂与顺铂治疗NSCLC的生存率 The survival rate of gecitabine+cisplatin versus cisplatin

##### 2.3.2.3 不良反应

###### 2.3.2.3.1 血液学不良反应

1个研究^[12]^报告了3/4级血液性毒副反应发生率，结果显示：与单用顺铂相比，吉西他滨联合铂类的血小板减少症发生率（OR=28.55, 95%CI: 14.06-57.04, *P*=0.000 01）、中性粒细胞减少症发生率（OR=27.53, 95%CI: 14.68-51.64, *P*=0.000 01）以及贫血发生率明显增加（OR=4.80, 95%CI: 2.73-8.46, *P*=0.000 01）（图 9）。

**9:**
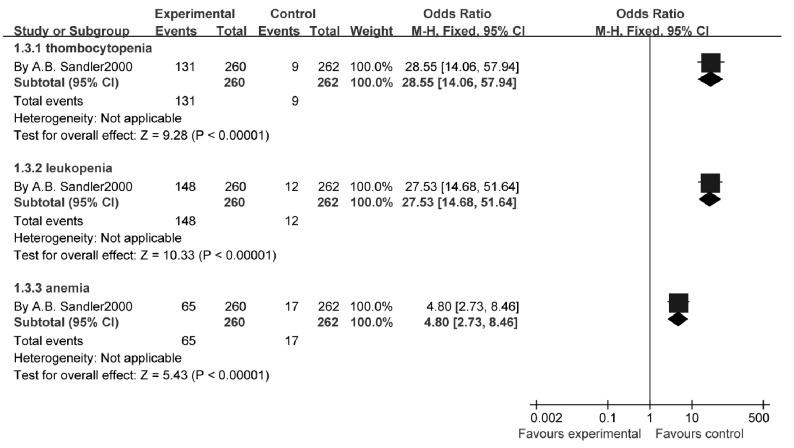
吉西他滨联合卡铂与顺铂治疗NSCLC的血液学不良反应 The hemotologic toxicity rate of gecitabine+carboplatin versus cisplatin

###### 2.3.2.3.2 非血液学不良反应

1个研究^[12]^结果显示：与单用顺铂相比，吉西他滨联合铂类的消化道不良反应（恶心/呕吐、便秘/腹泻）发生率明显增加，差异有统计学意义（OR=1.66, 95%CI: 1.18-2.35, *P*=0.004），但不增加肺相关不良反应呼吸困难的发生率（OR=1.42, 95%CI: 0.68-2.97, *P*=0.35）（图 10）。

**10:**
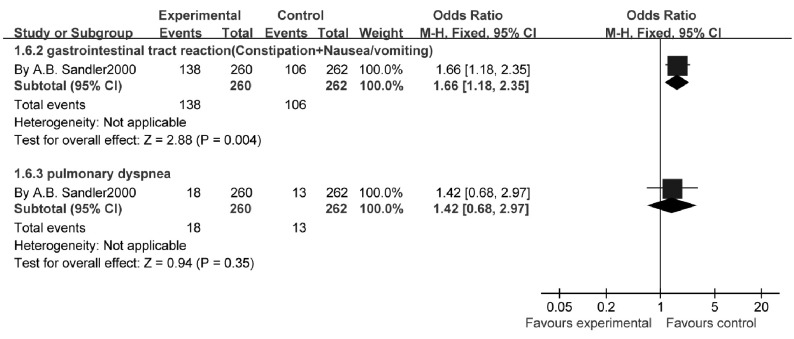
吉西他滨联合卡铂与顺铂治疗NSCLC的非血液性不良反应 The unhemotologic toxicity rate of gecitabine+carboplatin versus cisplatin

## 3 讨论

吉西他滨为细胞周期特异性抗肿瘤药物，仅对增殖周期的某些时相敏感（S期）而对G_0_期细胞不敏感，在体内需要一定时间才能发挥杀伤作用，剂量反应曲线是一条渐近线，即小剂量时类似于直线，达到一定剂量时效应不再增加。铂类为细胞周期非特异性抗肿瘤药物，能杀灭处于增殖周期各时相甚至包括G_0_期细胞的药物，在体内可迅速杀死肿瘤细胞，剂量反应曲线接近直线，在体内能耐受的毒性范围内，其杀伤能力随剂量的增加而成倍增加。吉西他滨或铂类单用与吉西他滨联合铂类方案在治疗NSCLC的疗效和安全性方面是否存在差异是颇受关注的问题。

本次*meta*分析结果显示，与单用吉西他滨相比，吉西他滨联合铂类可明显提高有效率和2年生存率，但同时也增加了3/4级血小板减少的发生率和中性粒细胞减少的发生率，而1年生存率和3/4级消化道及呼吸系统不良反应发生率并无差异；与单用顺铂相比，吉西他滨联合顺铂可明显提高有效率和1年生存率，同时使3/4级血小板减少发生率、中性粒细胞减少发生率、贫血发生率、3/4级消化道不良反应发生率明显增加；3/4级呼吸系统不良反应发生率无差异。由于只纳入了1篇研究，而且研究对象为60岁以上的老年人，故在应用本结果时需要慎重。

综上所述，吉西他滨联合顺铂/卡铂与单用顺铂或吉西他滨相比，可明显提高NSCLC的有效性，但因不良反应发生率增加显著，其安全性受到质疑。因此，在将吉西他滨联合铂类作为NSCLC的化疗方案时应权衡利弊，充分考虑其毒副作用，监测患者血常规及各项生化指标，及时调整剂量，以减少不良反应的发生，提高患者生存质量和无症状生存时间。

本次*meta*分析纳入的4个研究，质量差异较大，存在一定的方法学缺陷，比如2个研究提及盲法但未具体描述，2个研究未提及盲法；1个研究虽标明“随机”，但未说明具体的随机方法；2个研究未提及分配隐藏等。由于纳入分析的RCT数量偏少，且质量差异较大，存在选择性偏倚、实施偏倚及测量偏倚的可能性大，对临床NSCLC病人治疗方案的确定指导意义不够。今后，尚需收集更多高质量的RCT的研究结果，对治疗NSCLC的疗效和安全性开展更为科学的评价。
